# Ultra-sensitive detection of 4-chloro-2-methylphenoxyacetic acid herbicide using a porous Co-1,4-benzenedicarboxylate /montmorillonite nanocomposite sensor

**DOI:** 10.1007/s00604-024-06765-8

**Published:** 2024-12-24

**Authors:** Mona Elfiky, Moa’mena Abdo, Mona Darwesh, Nehal Salahuddin

**Affiliations:** 1 Chemistry Department, Faculty of Science, Tanta University, Tanta, Egypt; 2 Basic Sciences Department, the Higher Institute of Engineering, Kafr El-Sheikh, Egypt; 3 Physics Mathematical Engineering Department, Faculty of Engineering, Tanta University, Tanta, Egypt

**Keywords:** 4-Chloro-2-methylphenoxyacetic acid, Metal–organic framework, Montmorillonite, Stripping voltammetry, Modified carbon paste electrode, Herbicide, Environmental monitoring

## Abstract

The detection of 4-chloro-2-methylphenoxyacetic acid (CMPA) herbicide is crucial due to the potential health risks linked to exposure through drinking water, air, and food, which may adversely affect liver and kidney functions. To address this environmental concern and promote sustainable agriculture, a sensitive carbon paste sensor incorporating a composite material was developed. The composite sensor is based on porous cobalt-1,4-benzenedicarboxylate metal–organic framework and exfoliated montmorillonite nanolayers (Co-OF/MMt). This sensor enables the voltammetric detection of CMPA in real soil samples using linear sweep adsorptive anodic stripping voltammetry (LS-AdASV), facilitating early and accurate monitoring of herbicide levels. The Co-OF/MMt nanocomposite was synthesized via a hydrothermal method involving the precipitation of Co-OF in the presence of MMt. Comprehensive characterization of the synthesized materials was carried out using Fourier transform infrared spectroscopy (FT-IR), X-ray powder diffraction (XRD), scanning electron microscopy (SEM), SEM-Energy dispersive X-ray spectroscopy (SEM–EDX) and surface area analysis (BET). The resulting modified carbon paste sensor, utilizing 1.0% Co-OF/MMt nanocomposite, exhibited superior electrochemical properties compared with the bare carbon paste sensor, possessing an electroactive surface area of 1004.1 m^2^/g with a minimal resistivity (R_ct_) of 330 Ω. Under standard operating conditions, the developed sensor demonstrated detection limits of 0.03 nM and 0.1 nM across two broad linear ranges (0.03 to 0.10 nM – 0.10 to 1.0 nM) and (0.1 to 1.0 nM – 1.0 to 7.0 nM), respectively, for CMPA determination in both bulk and soil samples. These results pointed out the promising electrochemical modified sensor for the direct and simple detection of certain herbicides in environmental matrices, without the need for sample pretreatment steps. This capability supports sustainable development goals by enhancing effective environmental monitoring.

**Graphical Abstract:**

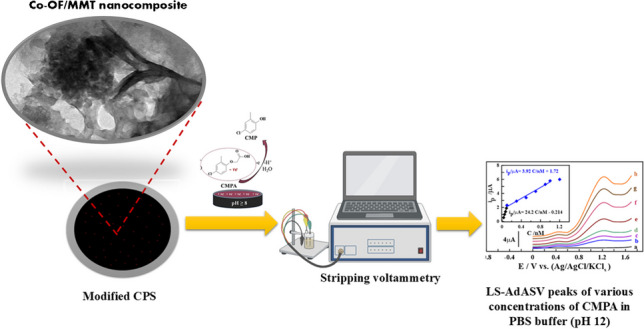

**Supplementary Information:**

The online version contains supplementary material available at 10.1007/s00604-024-06765-8.

## Introduction

Herbicides have become integral to modern agricultural practices globally. Nevertheless, despite their efficacy in pest management, these chemicals pose considerable hazards to natural ecosystems [1]. Among the widely used herbicides for managing broadleaf weeds in agricultural and horticultural crops is 4-chloro-2-methylphenoxyacetic acid (CMPA) (Scheme S1), which often contaminates groundwater sources [2]. CMPA acts as a selective herbicide targeting broadleaf weeds in various crops by emulating the action of auxins, natural plant growth hormones [2]. CMPA can be absorbed by plant roots and transported to leaves and stems through the phloem. The residual CMPA in soil usually persists with a half-life of around 24 days [3]. Given the widespread use of CMPA, concerns about its environmental impact have led to extensive research to assess its risks. CMPA exhibits moderate toxicity to mammals and aquatic organisms but is less toxic to birds [4]. However, detected concentrations of CMPA in water and soil generally fall below predicted no-effect levels across environmental compartments, suggesting a low risk [5, 6].

Various formulations of CMPA-based herbicides, including free acids, salts, and esters, release the acid as the active ingredient. Exposure to CMPA can occur through drinking water, air, food, and occupational settings related to pesticide application [7]. CMPA exposure can lead to symptoms such as nausea, vomiting, diarrhea, and abdominal pain, along with neurological effects like headaches, dizziness, and muscle weakness. High exposure levels may affect liver and kidney functions [8]. Regulatory limits set a maximum acceptable concentration of 0.35 mg/L (350 µg/L) based on observed kidney effects in rats [9]. Given the health and environmental concerns associated with CMPA, there is a pressing need for rapid, sensitive, and straightforward analysis methods. So far, several CMPA detection methods have been developed, and these including ultraviolet–visible (UV–Vis) spectroscopy, [10–14] high-performance liquid chromatography (HPLC), [15] liquid chromatography-tandem mass spectrometry, [16] capillary electrophoresis, [17] photocatalytic degradation, [18] solid phase extraction coupled with ion mobility spectrometry, [19] electrochemiluminescence [20] and chemiluminescence [21]. However, these methods suffer from expensive equipment, the requirement for trained technical personnel, complex sample preparation, and time-consuming analyses. Electrochemical sensors offer several advantages, including rapidity, reliability, cost-effectiveness, and portability, making them suitable for on-site and real-time detection [21–23].

While modified glassy carbon (MGC) sensors have been commonly used for CMPA detection. As displayed in (Table 1), the recent studies on CMPA assay achieved the limits of detection (LODs) of 0.008, 1.1, and 0.99 μM upon the simple activated GC sensor [21], a polyaniline/carbon nanotube (CNT) cyclodextrin matrix (PANI-β-CD/MWCNT)-based electrochemical sensor [24], and surface of combined β-CD with multi-walled carbon nanotubes (MWCNTs) modified GC sensor [25] in an aqueous phosphate solution, respectively. But they are costly and have a limited operational potential range compared to graphite paste (GP) sensors. However, bare GP sensor has a slow electron transfer rate, prompting the development of chemically modified GP sensors to enhance sensitivity in detecting CMPA in environmental samples.

**Table 1:** Presents a variety of analytical techniques and sensors employed in detecting CMPA

Developed Sensor	Volt. Teq	LR μM	LOD μM	Ref
Activated/GCE	DPV	1 – 850	0.008	[21]
SBA-15 CPE	CV	10 – 500	1.4	[22]
MIP/ SPE	SPC, potentiometry	0.04 – 1	0.01	[23]
PANI-β-CD/CNT/GCE	CV	10 – 50	1.1	[24]
PANI-β-CD/MWCNT/GCE	CV	10 – 100	0.99	[25]
Powdered activated carbon (Norit SX-2) paste	(DPV)	10 – 500	0.7	[22]
1.0% [Co-OF/MMt] CP sensor	LS-AdASV	0.03 – 0.1 nM (Bulk) 0.1 – 1.0 nM (Soil)	0.03	**This work**

Glassy carbon electrode (GCE), carbon paste electrode (CPE), mesoporous silica (SBA-15), differential pulse voltammetry (DPV), molecularly imprinted polymer (MIP), screen-printed electrode (SPE), polyaniline/carbon nanotube (CNT) cyclodextrin matrix (PANI-β-CD/MWCNT)

Metal–Organic Frameworks (MOFs) materials are employed in electrochemical applications as modifiers owing to their advantageous properties, including high porosity, extensive surface area, superior crystalline characteristics, stability, and tunable functionalities [26–32]. For instance, Li et al. [33], Yang et al. [34], developed a Co-OF nanosheet array sensors, which achieved low limits of detection (LODs) of 1.3 nM [33], 3.76 μM [34], and 1.85 μM [35] for detection of glucose, a hydrogen peroxide (H₂O₂), and L-cysteine, respectively. Nevertheless, many MOFs suffer from low conductivity, due to their inherently low electron transfer capabilities, which is essential for electrochemical sensing. But linear MOFs possess distinct advantages due to their long chains and interconnected one-dimensional channels, which facilitate efficient ion diffusion and enhance stability under various conditions [36]. The ligand-to-metal ratio critically influences electronic communication on MOF surfaces. As noted by Wang et al., a 2:1 ratio (H_3_BTC: Bi^3^⁺) in Bi-OF can improve metal ion connectivity but may hinder charge delocalization compared to a 1:1 ratio. In contrast, a 1:1 ratio (1,4-benzenedicarboxylate: metal) typically results in coordination polymers with well-defined geometries, promoting effective electronic communication and enhanced conductivity. Recent studies indicate that enhancing the conductivity of metal–organic frameworks (MOFs) through improved electron and charge transfer kinetics at the electrode/electrolyte interface can be effectively achieved by doping with various conductive materials. These include precious metals [37], carbon nanomaterials [38], carbon black [28], graphitic carbon nitride [29], and MOF-based composite materials [26, 30, 39].

Layered hydrous alumina-silicate clay (Montmorillonite, MMt) is the most prevalent smectite clay mineral, which has an irregular arrangement of lamellae structure with 100 nm in diameter and ∼1 nm in thickness and it is formed by two layers of tetrahedral silica bonded to an octahedral aluminum hydroxide layer. Exchangeable cations, such as Na^+^ in sodium-MMt, and water molecules can be found in the gaps between MMt layers. These substances have the ability to be modified by balancing out the negative charge produced on the lamellae via isomorphic substitution. MMt stacks were capable of incorporating guest species such as metal oxides, [40] metal–organic framework [41], and conducting polymers [42] to form hybrids or/ and nanocomposites as a new generation of materials with improved physical, and chemical properties for different types of applications. Despite these advancements, there is currently no detailed report on the fabrication of nanocomposites based on a porous Co-1,4-benzenedicarboxylate metal–organic framework with exfoliated montmorillonite nanosheets (Co-OF/MMt) for electrochemical sensing.

In this context, this study is based on the development of a novel Co-OF/MMt nanocomposite that integrates porous Co-OF with swelled montmorillonite nanosheets (Co-OF/MMt) via a single-step hydrothermal method. By addressing the conductivity limitations inherent in traditional modified graphite paste sensors, this hybrid material promises improved sensitivity and faster electron transfer rates, making it suitable for environmental monitoring applications. The unique structural advantages of linear Co-OF, coupled with the ability to incorporate various conductive materials like MMt clay, may lead to enhanced ion diffusion and stability, thereby facilitating real-time detection of CMPA at lower limits of detection in both bulk and soil samples than previously achieved. Furthermore, this advancement could pave the way for the creation of multifunctional sensing platforms capable of detecting multiple contaminants simultaneously, significantly contributing to environmental protection and public health initiatives.

## Experimental part

### Materials

Na-Montmorillonite (Na-MMt), obtained from Southern Clay Products (Colloid BP), Inc. (Gonzales, Texas, USA), has a cation exchange capacity of 114.8 meq/100g. The MMt clay underwent vacuum oven drying at 100°C for 24 h, resulting in an interlayer spacing (d_001_) of 9.6 Å. Cobalt (II) nitrate hexahydrate (Co(NO_3_)_2_.6H_2_O, 98%), terephthalic acid (BDC, 98%), potassium hexacyanoferrate (III) (K_3_[Fe(CN_6_)], 99.0%), ethanol (EtOH, 98%), N,N-dimethylformamide (DMF, 98%), phosphoric acid (H_3_PO_4_, 99.0%), sodium chloride (NaCl), sodium phosphate monobasic (NaH_2_PO_4_, 99.0%), and disodium hydrogen phosphate dihydrate (Na_2_HPO_4_.2H_2_O, 99.5%), sodium phosphate (Na_3_PO_4_, 96.0%), sodium hydroxide (NaOH pellets, 98.0%), potassium chloride (KCl, 99.0%), and 4-chloro-2-methylphenoxyacetic acid (CMPA) were purchased from Sigma-Aldrich and utilized without further purification.

### Structural characterization instruments

This part is detailed in the electronic supporting file.

### Preparation of electroanalytical liquors

A freshly prepared stock solution of CMPA (0.001 M) was diluted over the range of 10–0.001 µM using double-deionized water (DDW). Several phosphate-buffered saline (PBS) solutions were prepared by mixing varying proportions of 0.1 mol/L of Na_2_HPO_4_, NaH_2_PO_4_, and 0.1 mol/L NaOH in DDW, which were subsequently utilized as supporting electrolytes. Additionally, a stock solution of K_3_[Fe(CN)_6_] (1.0 mol/L) and 0.1 mol/L of KCl was freshly prepared as a redox probe for the cell system and employed in the electrochemical investigations of the fabricated sensor. Moreover, 0.5 g of the soil sample was spiked with different concentrations of CMPA in a micro-electrochemical cell (10 mL volume) containing 5.0 mL DDW and 5 mL buffer solution (pH 12), followed by sonication for 30 min and used for subsequent analytical measurements.

The pH drift process was utilized to determine the pH point of zero charge (pH_ZPC_) of the Co-OF/MMt nanocomposite [43]. Initially, 0.1 M HNO_3_ or NaOH was added to a series of 20 mL 0.01 M NaNO_3_ solutions under N_2_ atmosphere to adjust the initial pH values over the range of 1.5–10. Subsequently, 20 mL of each prepared NaNO_3_ solution was mixed with 0.06 g of Co-OF/MMt nanocomposite, and the mixture was gently shaken continuously for two days. Upon removal of the adsorbent by filtration, the final pH (pH_f_) was determined for the resulting filtrate solution. Thereafter, a plot of δpH (pH_f_ − pH_i_) versus pH_i_ was constructed to determine the pH_ZPC_ of the Co-OF/MMt nanocomposite.

### Synthesis of porous Co-OF and Co-OF/MMt nanocomposite

A porous Co-OF material was synthesized using a single hydrothermal method as follows: 2 g of Co(NO_3_)_2_.6H_2_O was dissolved in 17 mL of double deionized water (DDW) (beaker A), while 1 g of 1,4-benzene dicarboxylate acid (BDC) was dissolved in 34 mL of a mixture of N,N-dimethylformamide (DMF) and ethanol (Eth) (beaker B) [44]. Subsequently, the solution from beaker A was slowly added dropwise to beaker B under constant stirring, and the resulting mixture was allowed to react in an autoclave at 120°C for 24 h. The obtained precipitate was washed thoroughly with DMF and ethanol. Finally, the precipitate was dried in an oven at 80°C for 12 h, resulting in a yield of 2 g. A porous Co-OF/MMt nanocomposite was synthesized using the last-mentioned synthesis with the addition of 0.2 g of swelled MMT clay in (beaker A), resulting in a yield of 2.4 g.

### Fabrication of bare and modified sensors

A bare carbon paste (BCP) sensor was produced by combining 5 g of fine carbon powder with 1.8 mL of paraffin oil. The resulting carbon paste was then tightly packed into the sensor cavity with an inner diameter of 3.0 mm. Then, the sensor underwent polishing and was immersed in an electrolysis cell. The 1.0% [Co-OF/MMt] CP sensor was prepared by blending 4.95 g of fine graphite powder with 0.05 g of modifier powder in the presence of 1.8 mL of paraffin oil. Various amounts (0.5% and 2.0%) of the modifier powder were utilized to produce different CP sensors.

### Electrochemical measurements

The electrochemical measurements were carried out in a micro-electrochemical cell with a three-electrode system, including modified CP as the working sensor, Ag/AgCl/KCl as the reference electrode, and platinum wire as the auxiliary electrode. The electroactive surface area of all prepared MCPs was assessed using CV technique, detecting 1.0 mM K_3_[Fe(CN_6_)] with 0.1 M of KCl, applying potential range of -0.7 to 0.15V at scan rate of (*v*) 100 mV s^−1^. The LS-AdASV technique scans were performed using the chosen sensor in a micro-electrochemical cell (10 mL volume) containing a chosen amount of DMMP, DW and filled with a specific pH value under the chosen operational conditions. LS-AdASV voltammograms were recorded after applying the anodic potential range.

## Results and discussion

### Characterization of fabricated porous materials

**Fig. 1:**
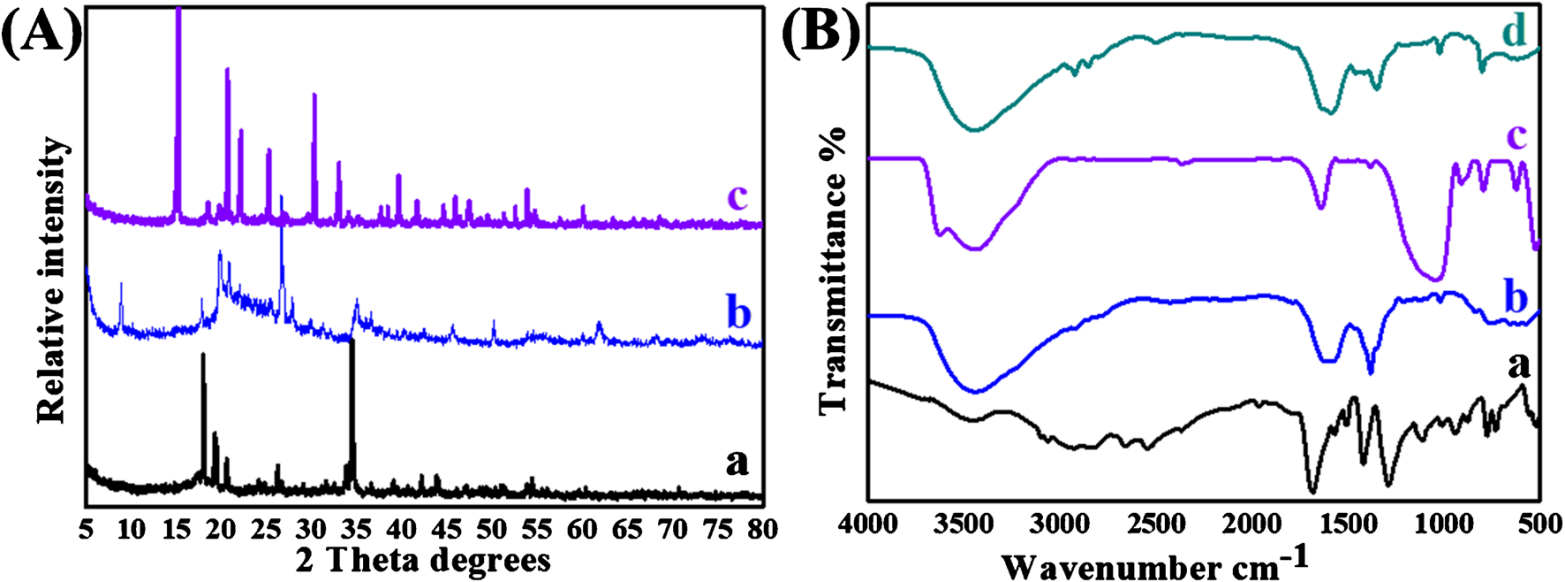
**A** The XRD pattern of (**a**) Co-OF, (**b**) MMt, and (**c**) Co-OF/MMt. **B** The FTIR spectra of (**a**) BDC, (**b**) Co-OF, (**c**) MMt and (**d**) Co-OF/MMt

The X-ray diffraction (XRD) patterns of Co-OF, MMt, and Co-OF/MMt are depicted in (Fig. 1A). In the XRD pattern of Co-OF (Fig. 1A_a_), the peaks align well with [JCPDS No. 96–901-2885] [45]. These peaks correspond to reflections from the 002, 111, 012, 201, 221, and 220 planes of Co, suggesting a crystalline size of 48.2 nm as determined by the Scherrer equation [46]. On the other hand, the XRD pattern of MMt (Fig. 1A_b_) exhibits a prominent peak at 8.8^◦^ attributed to the d_001_ plane. In the XRD pattern of Co-OF/MMt (Fig. 1A_c_), the disappearance of the crystalline peak corresponding to the d_001_ plane of MMt suggests the exfoliation of MMt sheets.

Furthermore, the Fourier transform infrared (FT-IR) spectra of BDC, Co-OF, MMt, and Co-OF/MMt are presented in (Fig. 1B) for structural analysis. The FT-IR spectrum of BDC (Fig. 1B_a_) exhibits characteristic bands around 1686, 1289, and 730 cm^−1^, attributed to the stretching *v*_C=O_, *v*_C–O_, and in-plane bending *v*_Ar–H_ [45], respectively**.** In contrast, the FT-IR spectrum of Co-OF (Fig. 1B_b_) displays a characteristic band at approximately 3448 cm^−1^, possibly corresponding to the stretching *v*_H–O–H,_ and *v*_OH_. bands were shifted from 1686 to 1587 cm^−1^, and the ν_COO_ stretching band was shifted from 1289 to 1384 cm^−1^ Notably, the characteristic band in-plane bending *v*_Ar–H_ band of BDC was shifted from 730 to 763 cm^−1^, indicates the successful formation of pure Co-OF [47, 48]. The FTIR spectrum of MMt (Fig. 1B_c_) reveals a stretching ν_Si–O_ band at 1056 cm^−1^, as well as broad bands at 3444, and a band at 1641 cm^−1^ attributed to the stretching of O–H group, deformation of H_2_O and interlayer water molecules on MMt [49], respectively. Additionally, bands at 1046, 524, and 468 cm^−1^ are assigned to the stretching of Si–O, Si–O–Al [50], and Si–O-Si, respectively [51]. In the FT-IR spectrum of Co-OF/MMt (Fig. 1B_d_), a shift in the characteristic bands of ν_COO_ stretching and in-plane bending *v*_Ar–H_ bands of BDC from 1587 to 1590 cm^−1^, 1384 to 1215 cm^−1^, and 763 to 803 cm^−1^, respectively, suggests successful bonding between Co-OF and exfoliated MMt clay.

**Fig. 2:**
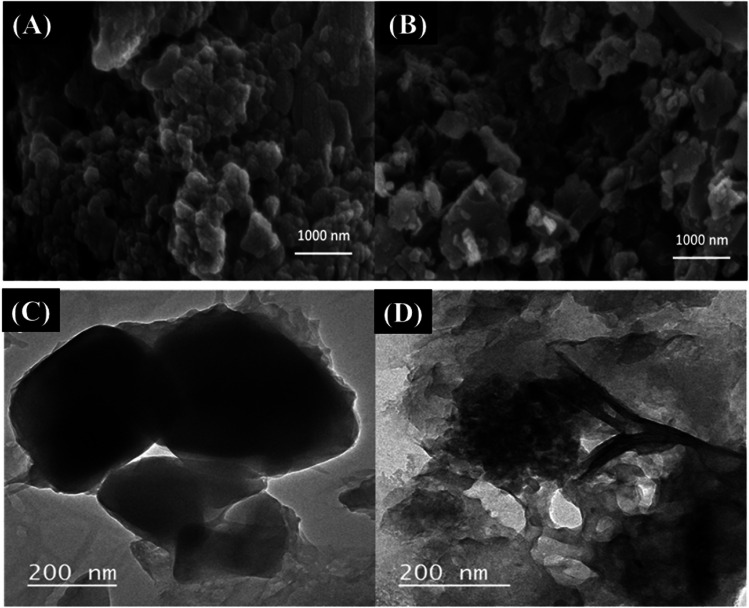
SEM micrograph of porous (**A**) Co-OF and (**B**) Co-OF/MMt materials. TEM micrograph of Co-OF (**C**) and Co-OF/MMt (**D**)

**Fig. 3:**
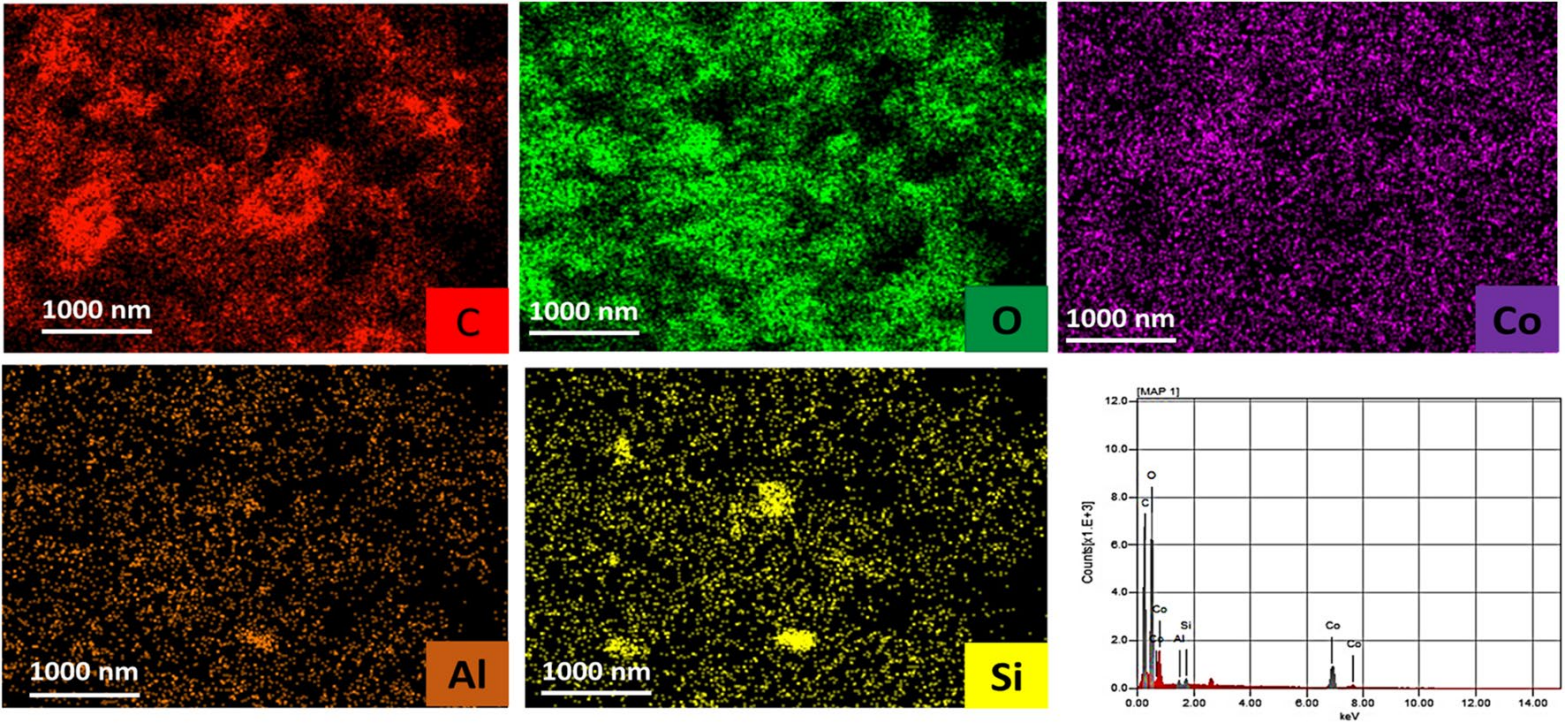
SEM–EDX elemental mapping distribution and mass percent spectrum of Co-OF/MMt

SEM, TEM, SEM–EDX, and BET analyses were carried out to explore the morphological properties of the prepared materials, as these properties are crucial for their performance. The SEM images of porous Co-OF (Fig. 2A) and Co-OF/MMt (Fig. 2B) materials revealed uniform and dense growth of porous Co-OF, even in the presence of peeled clay layers of MMt. Moreover, the SEM images of the porous Co-OF and Co-OF/MMt materials revealed average particle aggregate sizes of 260 nm and 300 nm (Image J software), respectively. Further investigation of the morphology of the porous Co-OF and Co-OF/MMt materials was carried out using TEM analysis, as depicted in (Fig. 2C, D). The TEM image of the porous Co-OF material exhibited a highly crystalline cubic morphology (Fig. 2C), with an average diameter of 170 nm. Notably, in (Fig. 2D), a uniform and dense growth of porous Co-OF was observed, even in the presence of peeled clay layers of MMt, with an average diameter of 10 nm (particle size of 150). Additionally, the SEM–EDX analysis provided elemental mapping of C, O, Al, Si, and Co in the Co-OF/MMt sample. This analysis confirms the incorporation of MMt clay layers and the uniform distribution of Co-OF within the clay layers, as illustrated in (Fig. 3). The spectrum reveals the presence of C, O, Al, Si, and Co within the structure, with mass percentages of 38.48%, 50.59%, 0.31%, 0.42%, and 10.20%, respectively.

Moreover, BET measurements indicated that Co-OF/MMt possessed the highest specific surface area (1004.1 m^2^/g) compared to Co-OF (378.6 m^2^/g). The increased BET specific surface area of Co-OF/MMt is anticipated to enhance electron and ion transport, thereby improving the electrochemical performance of the material.

**Scheme 1:**
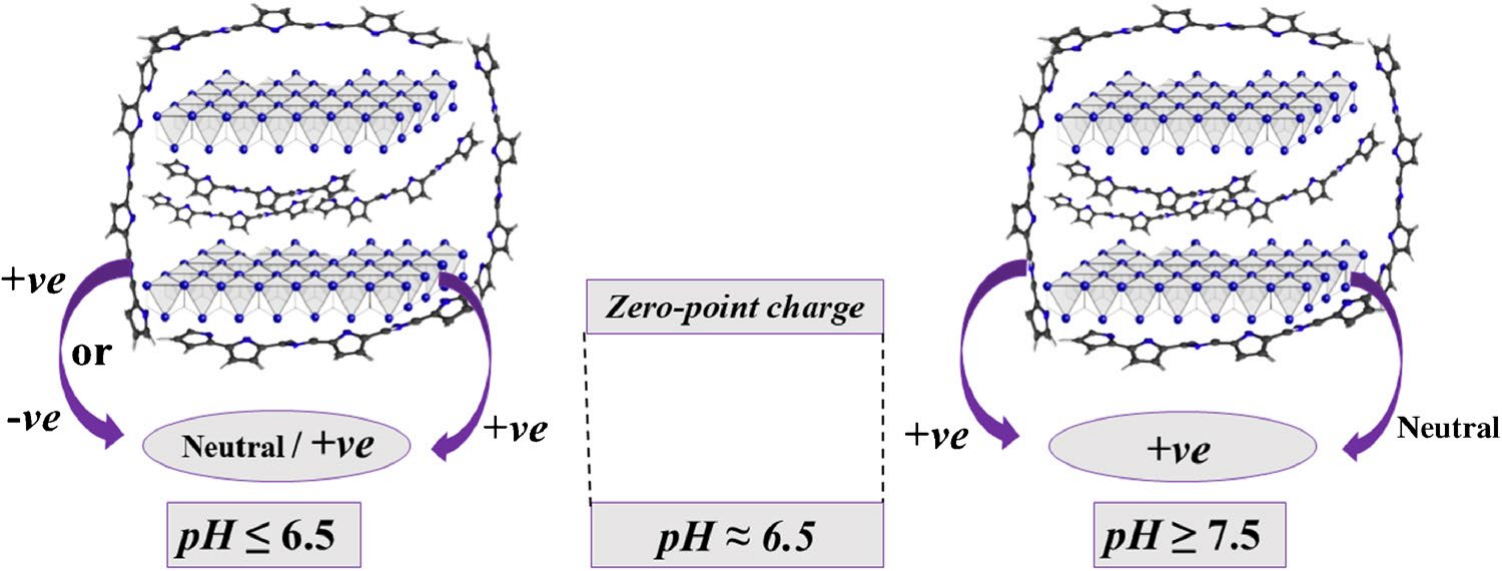
The net charge of the Co-OF/MMt composite in different pH values

As depicted in (Fig. S1), the point of zero charge (pH_PZC_) of the Co-OF/MMt composite, as prepared, is determined to be pH 6.3. In acidic conditions with a pH of ≤ 6.2, aluminum ions within the MMt structure may undergo protonation reactions. This occurs when Al(OH)₃ reacts with a proton (H⁺) to produce AlOH₂⁺, resulting in a positively charged aluminum species within the MMt structure [52]. This process is depicted in (Scheme 1). Additionally, the Co-OF can undergo both protonation (BDC + H⁺ ↔ HBDC) and deprotonation (HBDC ↔ BDC + H⁺) reactions. The balance between these reactions depends on the acidity level of the supporting medium used in the electrochemical sensing process, as indicated in (Scheme 1). If BDC becomes protonated (HBDC), the MOF may acquire a net positive charge due to the presence of H⁺. Conversely, if BDC undergoes deprotonation (resulting in BDC and releasing H⁺), the MOF may exhibit a net negative charge due to the presence of (-COO⁻) functional groups [53].

In basic conditions with a pH of ≥ 7.5, hydroxide ions (OH⁻) are able to interact with the positively charged aluminum species (AlOH₂⁺), leading to the formation of Al(OH)₂ and resulting in a neutral MMt structure, with water (H₂O) being released [52]. This process is depicted in (Scheme 1). Additionally, the Co-OF can undergo deprotonation reactions, where OH⁻ serves as a base by abstracting an H⁺ from the Co-OF. This results in the formation of a ligand-OH⁻ coordinated to the Co center and the release of an electron, thereby giving the MOF an overall positive charge, as indicated by the following equation:

$$\text{Co}-\text{OF }+ {\text{OH}}^{-} \to \text{ Co}-\text{OF}(\text{OH}) +\text{ e}^{-}$$

### Electrochemical characterization of as-prepared sensors

#### The preliminary stripping voltammetry test of as-prepared sensors

**Fig. 4:**
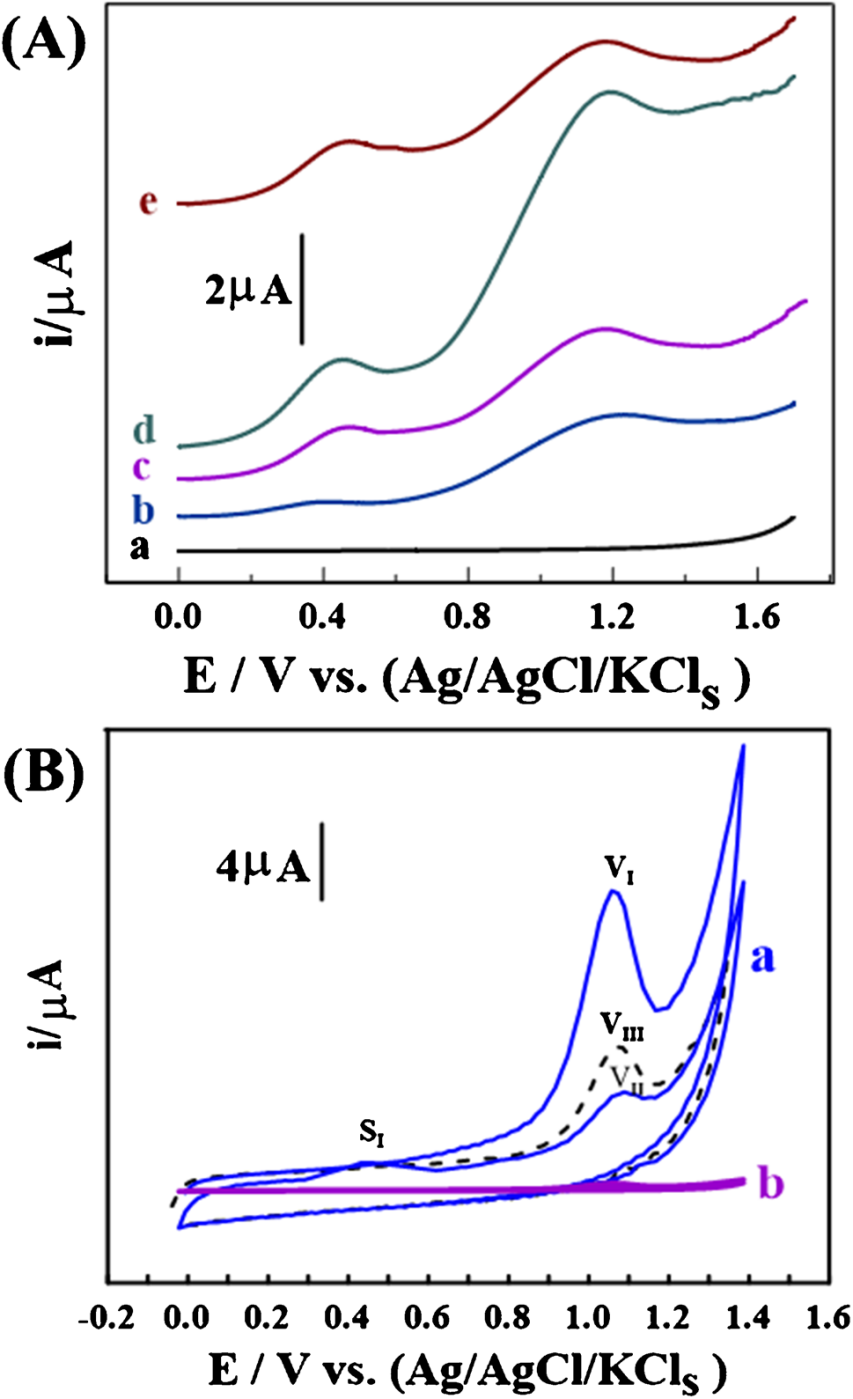
**A** Voltammograms for 0.5 nM CMPA at pH 12 were obtained using: (**a**) BCP, (**b**) 1.0% [Co-OF] CP, (**c**) 0.5% [Co-OF/MMt] CP, (**d**) 1.0% [Co-OF/MMt] CP, and (**e**) 2.0% [Co-OF/MMt] CP. **B** CV voltammograms for 1.0 nM CMPA at pH 12 were recorded with: (**a**) 1.0% [Co-OF/MMt] CP sensor (t_acc_ of 40 s; 1st cycle (V_I_), and 2nd cycle (V_II_)), and without time (V_III_)), and (b) BGCS (t_acc_ of 40 s; 2 cycles) at E_acc_ = 0.0V, and *v* = 100 mV.s^−1^

The LS-AdASV technique was utilized to initially detect 0.5 nM of CMPA in PBS buffer with a pH of 12, as shown in (Fig. 4A). The applied cumulative potential (E_acc_) and time (t_acc_) were 0.0 V and 50 s, respectively, with a scan rate (***v***) and scan increment (***ΔE***_s_) of 100 mV and 2.0 mV, respectively. As shown in (Fig. 4A), a 1.0% [Co-OF] CP sensor displayed an oxidation peak at 1.15 V (V_I_), corresponding to the cleavage of the ether linkage of CMPA to produce 4-chloro-2-methylphenol (CMP) as a byproduct, which also oxidized at 0.45 V (S_I_). Figures (Fig. 4A_a_, _b_), illustrate that the 1.0% [Co-OF] CP sensor exhibited distinct peaks compared to a BCP sensor, attributed to the porous structure of the Co-OF material, facilitating electron transfer pathways on the sensor surface. Furthermore, the 1.0% [Co-OF/MMt] CP sensor demonstrated the highest peak current intensity (i_p_) response for CMPA detection compared to 0.5% and 2.0% [Co-OF/MMt] CP sensors, exhibiting approximately a threefold increase relative to the 1.0% [Co-OF] CP sensor, as illustrated in (Figs. 4A_b_, _d_). This enhancement may be attributed to the increased electroactive surface area resulting from improvements in the structural integrity and distribution of active sites on the sensor's surface. These enhancements prevent excessive aggregation and ensure a more uniform distribution of the electroactive sample on the surface of sensor. In contrast, the 2.0% [Co-OF] CP sensor exhibited a decrease in peak current intensity, likely due to the aggregation of CMPA molecules into layered formations. These aggregates may subsequently re-dissolve into the solution before detection, thereby compromising the effectiveness of the electrochemical analysis [54].

#### Electrochemical behavior of CMPA and sensor mechanistic reaction

To elucidate the electrochemical characteristics of CMPA, cyclic voltammograms (CVs) were conducted for 1.0 µM CMPA in various PBS buffer solutions using the 1.0% [Co-OF/MMt] CP sensor, as depicted in (Fig. S2A). In the forward scan, a distinct oxidation peak at 1.15 V (V_I_) associated with the cleavage of the ether linkage of CMPA was observed, with no corresponding peak in the reverse direction, indicating the irreversibility of CMPA oxidation.

**Scheme 2:**
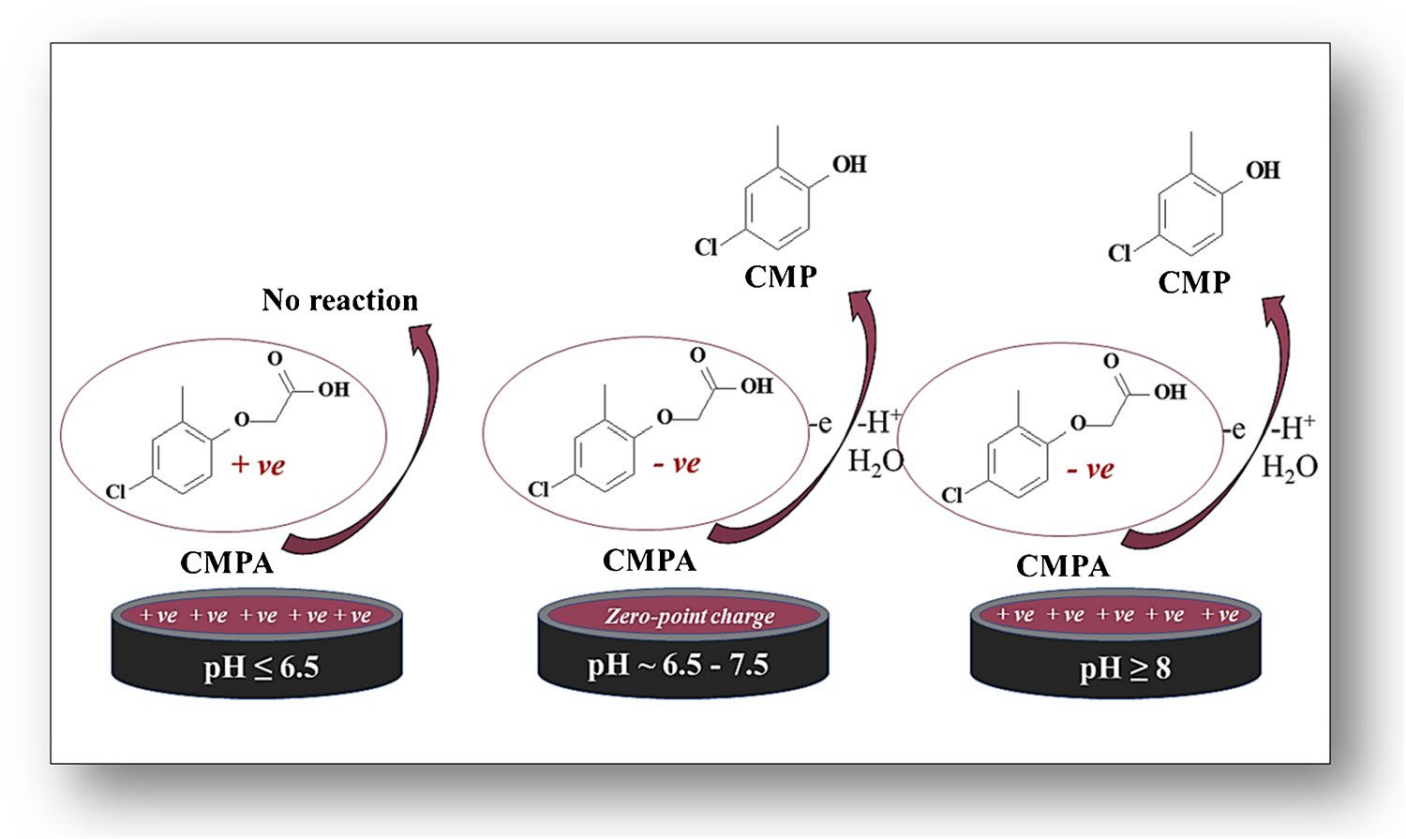
The suggested mechanism of CMPA on the surface of 1.0% [Co-OF/MMt] CP sensor

As proposed in (Scheme 2), the phenoxy acetic acid (Ar–O-COOH) functional group of CMPA interacted with the surface of the 1.0% [Co-OF/MMt] CP sensor via electrostatic attraction, governed by Lewis acid–base interactions. Figure (Fig. S2B) confirms a gradual rise in peak current intensity of voltammograms from pH 6 to 12, with the highest i_p_ achieved at pH 12. Below pH 6.0, no oxidation peak emerged due to repulsion between the strongly protonated form of CMPA molecules (+ *νe*) in the acidic medium (pK_a_ ~ 4.5) and the positively charged surface (+ *νe*) of the 1.0% [Co-OF/MMt] CP sensor (pH_zpc_ ~ 6.3; (Fig.S1)), as illustrated in (Schemes 1, 2). Conversely, at pH ≥ 6.5, the i_p_ became evident due to attraction between the lone pair of electrons on the phenoxy groups (-*νe*) of deprotonated CMPA molecules (-*νe*) (pK_a_ ~ 4.5) and the positively charged surface (+ *νe*) of the 1.0% [Co-OF/MMt] CP sensor. Hence, the reaction mechanism predominantly relies on electrostatic attraction alongside adsorption characteristics between CMPA molecules and the surface of the 1.0% [Co-OF/MMt] CP sensor.

Moreover, CVs of CMPA at the developed sensor were recorded in different pH values at *v* of 100 mV.s^−1^, as shown in (Fig. S2C). A linear negative shift in E_p_ with increasing pH was observed, following the equation: E_p_/V = (-0.054 ± 0.015) pH + (1.69 ± 0.46) (R^2^ = 0.99 and *n* = 5) (pH of 5.0–12.0), indicating proton involvement in the electrochemical oxidation of CMPA at the surface of the 1.0% [Co-OF/MMt] CP sensor. The slope value of 0.054 V/pH closely approximates the Nernst slope of 0.059 V/pH at 25°C, suggesting equal participation of protons and electrons in the oxidation process of CMPA. According to a previous report [24], the proposed oxidation mechanism of CMPA occurs at the aromatic ring, involving electron loss to form a cation radical intermediate, which leads to cleavage of the ether linkage of CMPA and the production of 4-chloro-2-methylphenol (CMP) as a byproduct, as illustrated in (Scheme 2).

Conversely, a linear correlation between Log i_p_ and Log ν in PBS buffer at pH 12 was established to explore the adsorption-controlled mechanism of CMPA using the 1.0% [Co-OF/MMt] CP sensor. This relationship is represented by the equation: Log i_p_/μA = (1.139 ± 0.56) Log ν/mV·s^−1^ + (1.66 ± 0.76) (*n* = 7 and R^2^ = 0.995), as depicted in (Fig. S2D). The calculated slope indicates that the oxidation of CMPA on the surface of the 1.0% [Co-OF/MMt] CP sensor is governed by the adsorption process. Furthermore, CVs of 1.0 nM CMPA in PBS buffer at pH 12 were conducted to assess the adsorption behavior of CMPA on both the 1.0% [Co-OF/MMt] CP sensor (Fig. 4B_a_) and BCP sensor (Fig. 4B_b_). The peak current (V_III_) of the voltammogram was recorded without utilizing an adsorptive accumulation time (under open circuit conditions). Subsequently, the peak current of voltammograms (V_I_) representing the initial cycle and (V_II_) representing the second cycle were recorded with an adsorptive accumulation time of 40 s.

#### Electroactive surface area and degree of resistivity properties

**Fig. 5:**
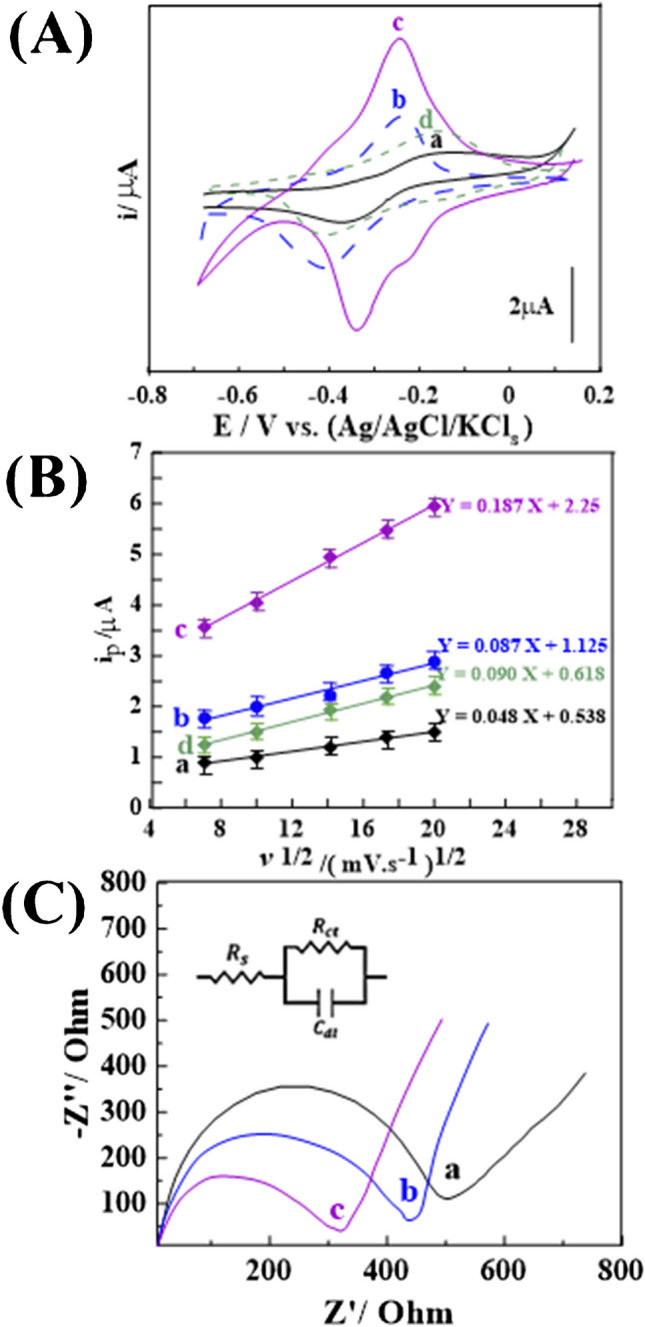
**A** CV voltammograms were recorded for 1.0 mM K_3_[Fe(CN_6_)] in 0.1 M KCl at 100 mV·s^−1^. **B** The I_p_ and ν^1/2^ plot from CVs at scan rates of 50 to 400 mV·s^−1^ is shown for (**a**) BGPS and (**b**) 0.5%, (**c**) 1.0%, and (**d**) 2.0% [Co-OF/MMt] CP sensors (*n* = 3). (**C**) Nyquist plots for 1.0 µM CMPA at pH 12 were generated using (**a**) BCP, (**b**) 1.0% [Co-OF] CP, and (**c**) 1.0% [Co-OF/MMt] CP sensors

Consideration should be given to the electroactive surface area, electron transfer rate (resistivity; R_ct_), and absorption properties when developing an ultra-sensitive CP sensor, as displayed in (Fig. 5). In (Fig. 5A), CV voltammograms of 1.0 mM K_3_[Fe(CN)_6_] in 0.1 mol.L^−1^ KCl as a redox probe material were recorded at *v* of 100 mV.S^−1^ using the BCP sensor and 0.5%, 1.0%, and 2.0% [Co-OF/MMt] CP sensors to evaluate their electroactive surface areas. The CV voltammograms revealed distinct redox peaks, indicating reversible electron transfer rates of the Fe^II^/Fe^III^ redox reaction compared to the BCP sensor. Additionally, the redox peaks exhibited *ΔE*_*p*_ values of approximately 0.14, 0.07, 0.06, and 0.24 V for the BCP sensor, and 0.5%, 1.0%, and 2.0% [Co-OF/MMt] CP sensors, respectively.

These *ΔE*_p_ values exceeded the expected theoretical value of 0.059 V associated with a fast one-electron transport, suggesting a quasi-reversible electron-transfer process. The smallest Δ*E*_p_, indicating the fastest electron-transfer kinetics at the sensor surface, was observed in the 1.0% [Co-OF/MMt] CP sensor. A well-defined redox peak of Fe^II^/Fe^III^ was evident in the 1.0% [Co-OF/MMt] CP sensor, indicating a larger electroactive surface area compared to the other sensors.

Furthermore, the electroactive surface area (A_s_) of all sensors was determined from the peak intensity (i_p_) of the CVs plotted against ν^1/2^ at v ≈ 0.20 – 4.0 V.s^−1^ (Fig. 5B). This correlated with the Randles–Sevcik Eq. (1) [55]:

1 $${i}_{\text{p}} = (2.69 \times {10}^{5}) {n}^{3/2} {A}_{\text{s}} {D}^{1/2} {v}^{1/2}$$

where D, C, and n represent the diffusion coefficient (7.6 µcm^2^·s^−1^), concentration of K_3_[Fe(CN)_6_], and number of electron transfers through the electrochemical process, respectively. The electroactive area values of the BCP, 0.5%, 1.0%, and 2.0% [Co-OF/MMt] CP sensors were calculated to be 0.065, 0.117, 0.253, and 0.121 cm^2^, respectively. Notably, the A_s_ of the 1.0% [Co-OF/MMt] CP sensor showed a twofold increase compared to the BCP sensor. This enhancement in A_s_ values of the CP sensor may be attributed to the morphological structure and specific surface area, as observed in TEM and BET analysis of the Co-OF/MMt material (Fig. 3D).

Alternatively, Electrochemical Impedance Spectroscopy (EIS) measurements were performed using Nyquist plots with a 1.0 μM CMPA solution in PBS buffer at pH 12, over a frequency range of 0.1 to 10,000 H_z_. These measurements were employed to elucidate the variations in electron transfer rates of the BCP and CP sensors during analyte detection, as shown in (Fig. 5C). The R_ct_ values of the BCP, 1.0% [Co-OF], and 1.0% [Co-OF/MMt] CP sensors were determined to be 500, 430, and 330 Ω, respectively. Notably, the 1.0% [Co-OF/MMt] CP sensor exhibited the lowest resistivity among all prepared sensors. Thus, it can be inferred that the 1.0% [Co-OF/MMt] CP sensor possesses distinctive surface properties, characterized by excellent A_s_ and *ΔE*_*p*_ values and minimal R_ct_, making it suitable for electroanalytical applications.

### Optimization of the instrumental and stripping parameters

The nature and concentration of the supporting electrolyte are crucial factors affecting the voltametric performance in detecting CMPA during the electrochemical interaction between the electroactive species and the sensor's surface. Thus, the voltametric response to 0.5 nM CMPA at the 1.0% [Co-OF/MMt] CP sensor was recorded in various PBS buffers ranging from pH 2 to 12, employing accumulation parameters (E_acc_ = 0.0 V and t_acc_ = 50 s). As depicted in (Fig. S3A), the highest and most distinct peak current response was observed at pH 12, thus chosen as the optimal pH level for CMPA detection.

Additionally, a series of anodic stripping voltammetric measurements were conducted on 0.5 nM CMPA in PBS buffer at pH 12 using the 1.0% [Co-OF/MMt] CP sensor to determine the optimal instrumental parameters, including varying *v* from 40 to 120 mV·s^−1^ (Fig. S3B), and *ΔE*_*s*_ from 2 to 12 mV (Fig. S3C). In conclusion, the optimum instrumental parameters for CMPA detection were identified as pH 12, ***v*** = 120 mV. S^−1^ and ΔE_s_ = 2 mV.

Notably, it is essential to monitor the concentration of the basic medium of the supporting electrolyte, ensuring it does not exceed 0.1 M. As, the prolonged exposure of Co-OF to high concentrations of hydroxide ions can lead to the formation of cobalt hydroxide Co(OH)_3_​. This reaction occurs as hydroxide ions deprotonate carboxyl groups in Co-OF, converting them into carboxylate anions and water:

$$\text{Co}-\text{OF}-\text{R}-\text{COOH}+{\text{OH}}^{-}\to \text{Co}-\text{OF}-\text{R}-{\text{COO}}^{-}+{\text{H}}_{2}\text{O}$$

In a strongly alkaline medium more than 0.1 M, the deprotonated Co-MOF may further react to form (Co(OH)₃) as follows:

$$\text{Co}-\text{MOF}-\text{R}-{\text{COO}}^{-}+{3\text{OH}}^{-}\to \text{Co}-\text{MOF}-\text{R}-{\text{COO}}^{-}+\text{Co}{(\text{OH})}_{3}$$

This conversion reduces lattice volume and enhances specific surface area due to "crystal-crystal conversion," leading to particle contraction, new micropore formation, and enlarged existing pores [56]. This process causes substantial particle contraction, formation of new micropores, and enlargement of initial pores, leading to an increased average pore diameter, total pore volume, and enhanced specific surface area. Moreover, Prolonged exposure to high hydroxide concentrations (pH 12) can destabilize montmorillonite, as the exchange of stabilizing cations (e.g., Na^+^, Ca^2+^) with hydroxide ions weakens its layered structure, increasing interlayer spacing and causing potential instability.

**Fig. 6:**
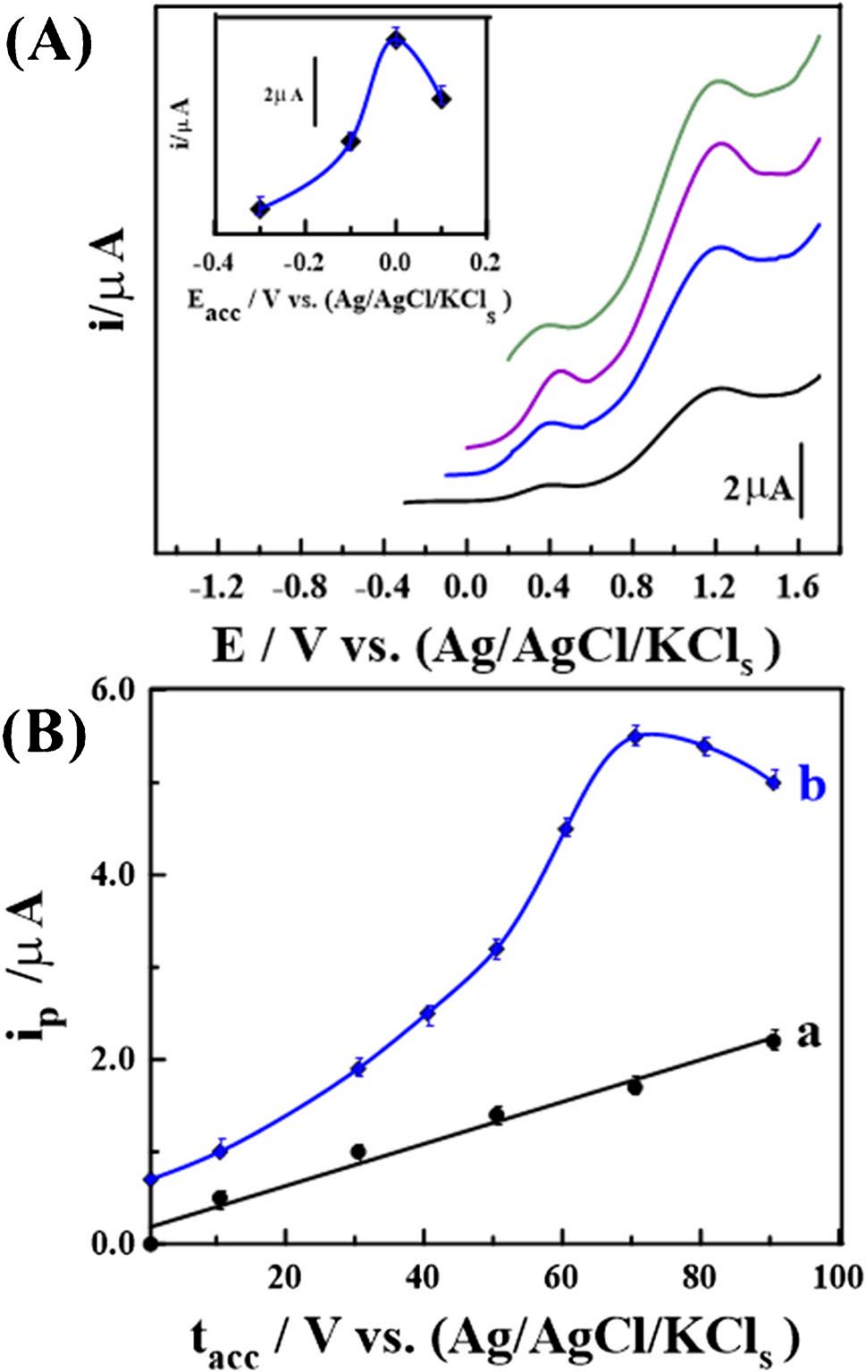
**A** Effect of varing of *E*_*acc*_ of 0.5 nM CMPA in (PBS buffer; pH 12) using 1.0% [Co-OF/MMt] CP sensor (*t*_*acc*_ = 50 s, *v* = 120 mV. S^−1^, *ΔE*_*s*_ = 2.0 mV). **B** the effect of changing *t*_*acc*_ of (**a**) 0.07, and (**b**) 0.5 nM CMPA at *E*_*acc*_ = 0.0 V (error bars for *n* = 3)

Furthermore, the accumulation step (stripping process) was found to significantly enhance the detection sensitivity for a sensor reaction driven by adsorption. Therefore, the impact of varying E_acc_ on the response of anodic peak current of 0.5 nM CMPA in PBS buffer at pH 12 on the surface of the 1.0% [Co-OF/MMt] CP sensor was assessed, as displayed in (Figs. 6A, B). As demonstrated in (Fig. 6A), a notably increased peak current was observed at E_acc_ of 0.0 V for t_acc_ = 50 s.

Similarly, the effect of varying t_acc_ on the magnitude of the anodic peak current for 0.07 and 0.5 nM CMPA in PBS buffer at pH 12 on the surface of the 1.0% [Co-OF/MMt] CP sensor was examined. As illustrated in (Fig. 6B), the anodic peak current magnitude was straight line, which gradually increased until reaching 90 s for 0.07 nM and 70 s for 0.5 nM, with a subsequent decline due to sensor saturation with CMPA. Consequently, the optimal accumulation parameters were determined to be E_acc_ = 0.0 V and t_acc_ = 50 s.

### The validity of electroanalytical determination of CMPA

#### The linearity range, and limit of detection

**Fig. 7:**
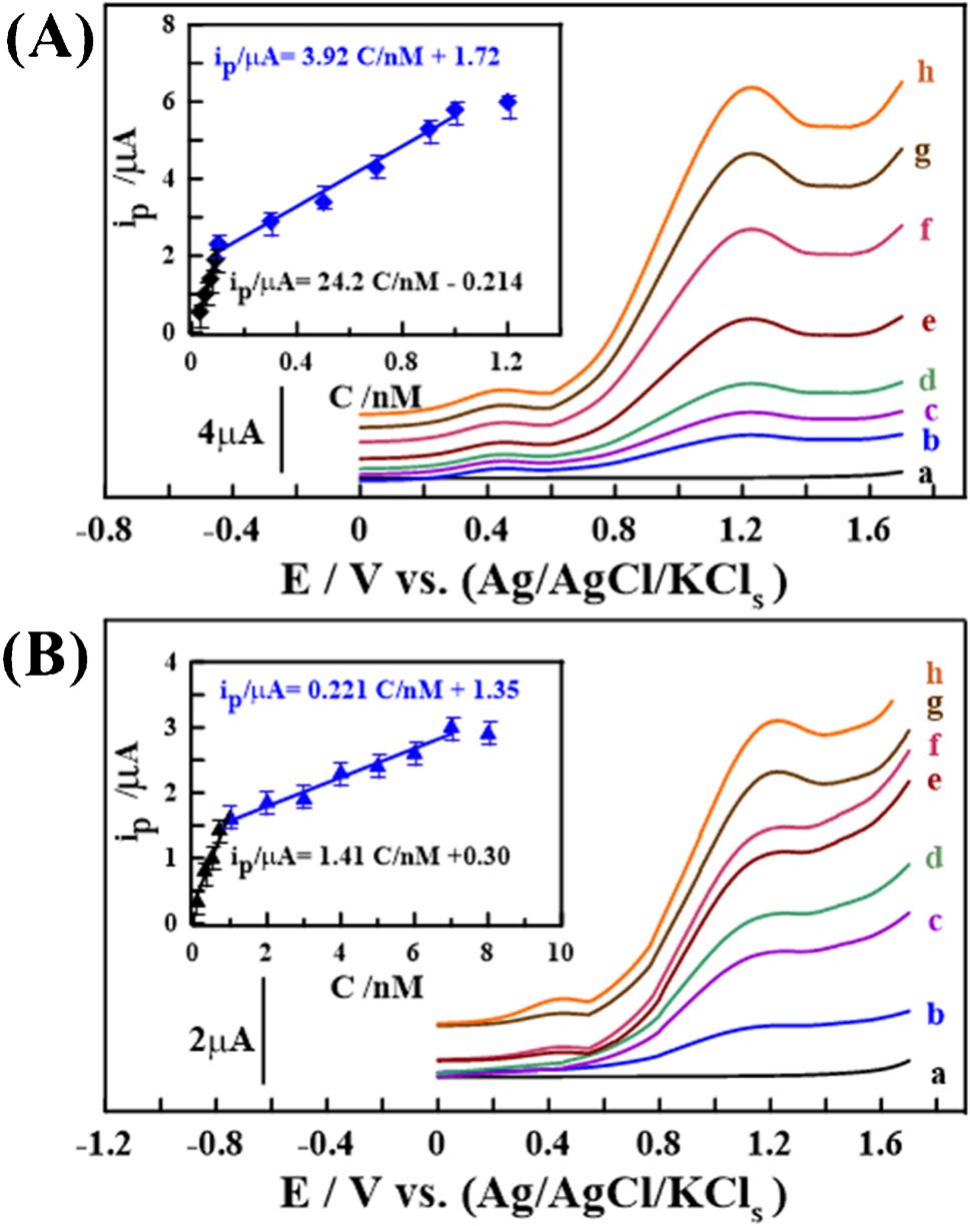
LS-AdASV peaks were observed for various CMPA concentrations in PBS buffer at pH 12 using a 1.0% [Co-OF/MMt] CP sensor (t_acc_ = 50 s, *v* = 120 mV·s^−1^, and *ΔE*_*s*_ = 2.0 mV). In **(A)**, the measurements were conducted in bulk form with concentrations ranging from (a) baseline to (h) 1.0 nM, with plots in the inset (*n* = 3). In **(B)**, measurements in spiked urine samples ranged from baseline to 7.0 nM, with plots in the inset (*n* = 3)

Using the optimal analytical parameters, the LS-AdASV method was validated by assessing various analytical aspects, including accuracy, precision, limit of detection (LOD), and the linearity range of the calibration curve. As depicted in (Fig. 7A), the calibration voltammograms and plots for CMPA determination in PBS buffer at pH 12 on the surface of a 1.0% [Co-OF/MMt] CP sensor were obtained, with linearity ranges (LR) of 0.03 to 0.1 nM and 0.1 to 1.0 nM, yielding sensitivity values (S/N = 3) of 24.18 and 3.91 μA.nM^−1^, respectively. The calibration plots were expressed by the following linear regression equations: i_p/μA_ = (24.17 ± 1.58) C_CMPA/nM_—(0.214 ± 0.053) (R^2^ = 0.994); and i_p/μA_ = (3.91 ± 0.54) C_CMPA/nM_ + (1.72 ± 0.58) (R^2^ = 0.991), with a LOD of approximately 0.03 nM. Several electroanalytical techniques and sensors have been developed for CMPA detection in various fluids, as summarized in (Table 1). From the information provided in (Table 1), it can be inferred that, compared to other analytical methods, the 1.0% [Co-OF/MMt] CP sensor offers the lowest LOD and the widest LR for CMPA determination in bulk and soil fluids. Additionally, there is limited research reports on the application of the LS-AdASV approach for soil CMPA detection.

#### Validity of proposed sensor

**Fig. 8:**
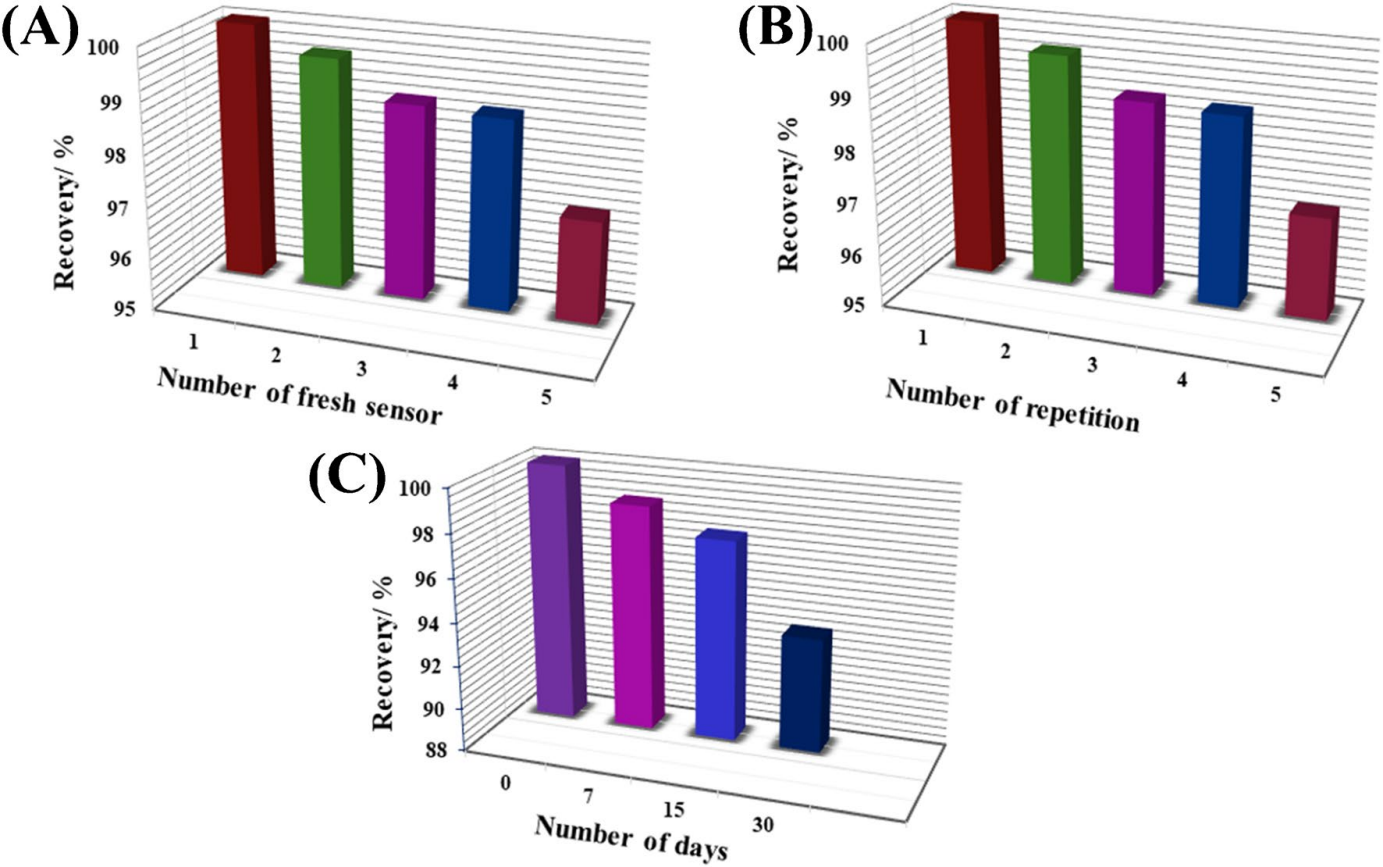
The reproducibility (**A**), repeatability (**B**), stability columns histogram of 0.3 nM CMPA upon the 1.0% [Co-OF/MMt] CP sensors

Under the specified optimal analytical conditions, the reliability and repeatability of the sensor were evaluated by conducting LS-AdASV voltammograms of 0.3 nM CMPA using five newly developed 1.0% [Co-OF/MMt] CP sensors simultaneously over the course of one day (intra-day analysis) and over three consecutive days (inter-day analysis), as illustrated in (Table 2). The analysis of CMPA demonstrated excellent reproducibility (Fig. 8(A)) and repeatability (Fig. 8(B)), with (recovery (R%) ± relative standard deviation (RSD)) of 98.67% ± 1.16 and 100.67% ± 2.54 for the intra-day and inter-day analyses, respectively. Furthermore, the stability of the 1.0% [Co-OF/MMt] CP sensor was evaluated three times (*n* = 3) every seven days for a period of one month while stored in ambient air at room temperature. The results indicate that the developed sensor retained approximately 97.33% of its initial activity after 15 days and maintained 93.3% activity after 30 days, as depicted in (Fig. 8(C)) and (Table 2).

**Table 2:** The intra- and inter-day analysis of 0.3 nM CMPA in the bulk form using the LS-AdASV method:

	*C*_Taken_(nM)	*C*_Found_(nM) ± SD	(R % ± RSD %)	Relative Error E_r_ (%)
*Intra-day analysis*	0.3	0.302 ± 0.008	100.67% ± 2.54	0.67
*Inter-day analysis*	0.3	0.296 ± 0.003	98.79% ± 1.16	-1.21
Stability _(n=3)_* 15 days 30 days	0.3	0.292 ± 0.004 0.280 ± 0.007	97.33 ± 1.80 93.3 ± 2.26	-2.67 -6.67

* (***n*** = 3) means three times of measurements

#### The interference property

The selectivity of the 1.0% [Co-OF/MMt] CP sensor was assessed by introducing common interferences typically found in soil fluid, as illustrated in (Fig. S4). Initially, the voltammogram of 0.3 nM CMPA (V_I_) (Fig. S4_a_) was recorded in (PBS buffer (pH 12)), followed by the addition of 30.0 nM (~ 100-fold) of common metal cations (Mix_1_: Mg^2+^, Zn^2+^, Co^2+^, K^+^, Ca^2+^, Na^+^, and Fe^2+^), and anions, such as (Mix_2_: SO_2_^2−^, PO_4_^3−^, CO_3_^2−^, and Cl^−^), as displayed in (Fig. S4_b_). It is noteworthy that no additional interferent peaks were observed for Mix1 or Mix2, and the voltammogram of 0.3 nM CMPA showed a negligible change in peak current magnitude, with an RSD of 1.41%. Additionally, (Fig. S4_c_) no interferent peaks were detected after adding 30.0 nM (~ 100-fold) glyphosate (GLYP) or chlorpyrifos (CPYP) near the CMPA peak in (PBS buffer (pH 12)), which is the optimal pH medium, despite differing from the reported optimized pH condition (PBS buffer (pH 7)) [44] for GLYP detection. Moreover, CPYP degradation occurred only in (PBS buffer (pH 2)) [45]. These findings indicate that even at concentrations exceeding 100-fold, the presence of common interferences in environmental samples such as soil and other insecticides/pesticides does not interfere with the detection of CMPA.

#### Application in environmental samples (soil fluid)

Under the carefully chosen optimal analytical conditions, LS-AdASV voltammograms and calibration plots were generated for the determination of CMPA in PBS buffer (pH 12) with the addition of spiked soil sample (Soil_1_) into electrochemical cell, along with the overall contents, eliminating the need for sample pre-treatment steps. As depicted in (Fig. 7B), the LR was from 0.1 to 1.0 nM (1.41 μA·nM^−1^) and 2.0 to 7.0 nM (0.22 μA·nM^−1^). The calibration plots yielded the following linear regression equations: i_p/μA_ = (1.41 ± 0.38) C_CMPA/nM_ + (0.30 ± 0.097) (R^2^ = 0.992); and i_p/μA_ = (0.221 ± 0.072) C_CMPA/nM_ + (1.35 ± 0.83) (R^2^ = 0.990), with a LOD of approximately 0.1 nM. Furthermore, LS-AdASV voltammograms of 0.7 and 2.0 nM CMPA were evaluated in the presence of two spiked soil samples (Soil_2_, _3_) obtained from different locations. These measurements exhibited satisfactory recovery (R%) ± relative standard deviation (RSD%) without interference from other soil components, as detailed in (Table 3). These outcomes highlighted the accuracy (RE%) and reliability of the developed sensor in detecting CMPA even in the presence of environmental interferences.

**Table 3:** Detection of CMPA in soil samples (*n* = 3)

Sample	C_Added_(nM)	C_Found_(nM) ± SD	R % ± RSD%	RE%
*Soil*_*2*_	0.70 2.00	0.689 ± 0.08 1.946 ± 0.35	98.40 ± 1.02 97.28 ± 2.53	-1.57 -2.72
*Soil*_*3*_	0.70 2.00	0.709 ± 0.005 2.036 ± 0.38	101.3 ± 3.31 101.8 ± 3.78	1.30 1.80

## Conclusion

A composite material comprising porous cobalt-1,4-benzenedicarboxylate metal–organic framework integrated with exfoliated montmorillonite was synthesized using a one-step hydrothermal process. Compared to the Co-OF material, this (Co-OF/MMt) nanocomposite exhibited an enhanced specific surface area of 1004.1 m^2^/g with crystalline size of 30 nm. Subsequently, a highly sensitive and reliable carbon paste sensor employing the Co-OF/MMt nanocomposite was developed for detecting CMPA in soil samples using LS-AdASV. Both cyclic voltammetry (CV) and electrochemical impedance spectroscopy (EIS) analyses of the CP sensor demonstrated superior electroactive surface area, electron transfer rates, and adsorption properties compared to the conventional BCP sensor. The resultant sensor demonstrates promising electrochemical characteristics, facilitating the sensitive and straightforward detection of certain herbicides and pesticides in real environmental samples, eliminating the need for elaborate sample pretreatment procedures.

## Supplementary Information

Below is the link to the electronic supplementary material.

Supplementary file1 (DOCX 378 KB)

## Data Availability

No datasets were generated or analysed during the current study.
