# Supplementary material for: Ultra-sensitive detection of 4-chloro-2-methylphenoxyacetic acid herbicide using a porous Co-1,4-benzenedicarboxylate /montmorillonite nanocomposite sensor
Source: Mikrochim Acta. 2024 Dec 24;192(1):30. doi: 10.1007/s00604-024-06765-8 (PMC11668838; doi:10.1007/s00604-024-06765-8)
Supplement: Supplementary file 1 — Supplementary file1 (DOCX 378 KB) [file 604_2024_6765_MOESM1_ESM.docx]

**Ultra-Sensitive Detection of 4-Chloro-2-Methylphenoxyacetic Acid Herbicide Using A Porous Co-1,4-Benzenedicarboxylate /Montmorillonite Nanocomposite Sensor**

Mona Elfiky ^a^*, Moa’mena Abdo ^b^, Mona. Darwesh ^c^, Nehal. Salahuddin ^a^*

^a^ Chemistry Department, Faculty of Science, Tanta University, Tanta, 31527 Egypt

^b^ Basic Sciences Department, the Higher Institute of Engineering, Kafr El-Sheikh, 31527 Egypt,

^c^ Physics Mathematical Engineering Department, Faculty of Engineering, Tanta University, Tanta, 31527, Egypt

^*^Corresponding author e-mail: Elfiky_mona@science.tanta.edu.eg, nehal.attaf@science.tanta.edu.eg

**
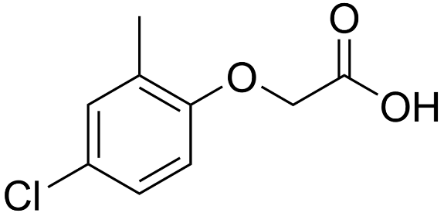
**

**Scheme S_1_:** Structure of 4-chloro-2-methylphenoxyacetic acid **(CMPA)**.

**2.2. The structural characterization instruments**

The surface characteristics of the synthesized nanocomposites were examined using a field emission scanning electron microscope (FE-SEM, Quanta TM 250) and high-resolution transmission electron microscopy (HR-TEM) JEM-2100 JEOL, employing a carbon-coated copper mesh grid (200 mesh). Fourier transform infrared (FT-IR) spectroscopy was conducted using a PerkinElmer spectrophotometer and a Rigaku Ultima IV R185. X-ray diffraction (XRD) analysis was performed with a Rigaku Ultima IV R185 X-ray diffractometer equipped with Cu-Kα (1.54A˚) at an acceleration voltage of 40 kV and a current of 20 mA. Additionally, the prepared nanocomposite underwent degassing at 150°C for 2 hours under vacuum before determining the specific surface area and pore size distribution using the Brunauer-Emmett-Teller (BET) method. Electrochemical impedance spectroscopy and stripping voltammetric analysis were conducted using a potentiostat (Model SI-1287, Solarton, Durham, UK) coupled with a frequency response analyzer (Model 1252A, Solarton) and a computer-controlled potentiostat (PAR) model 263 A, respectively.


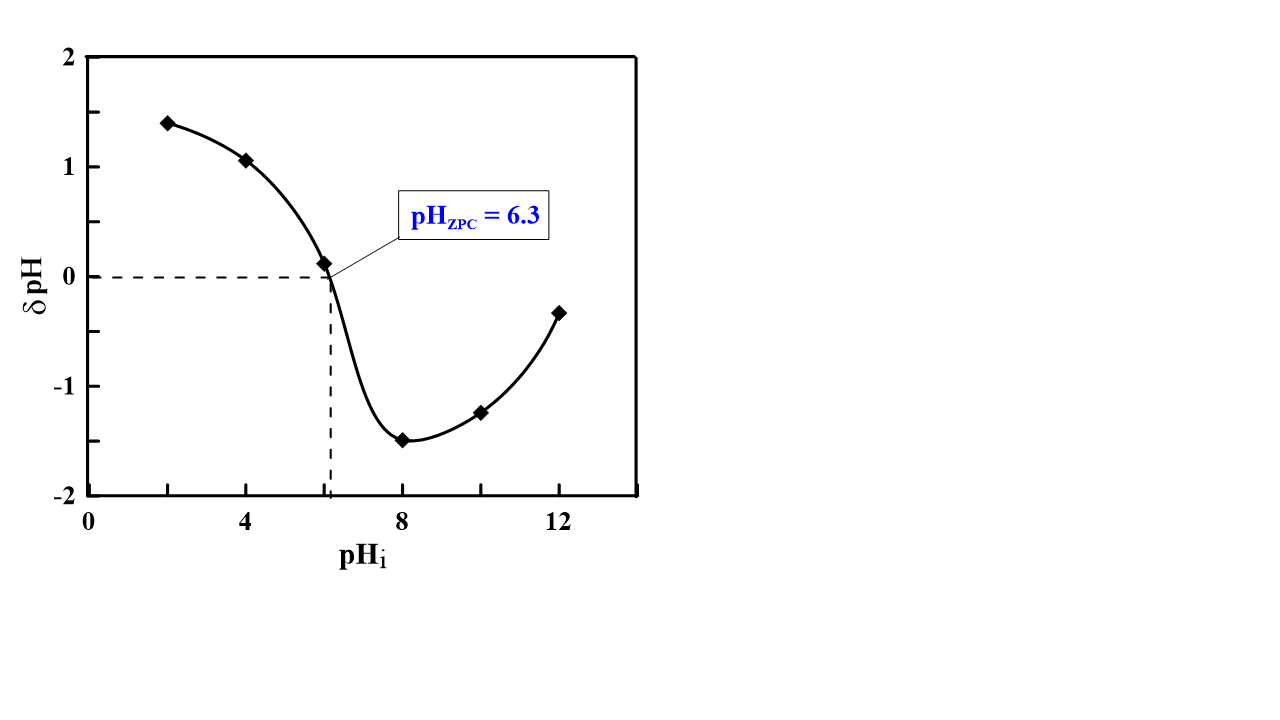


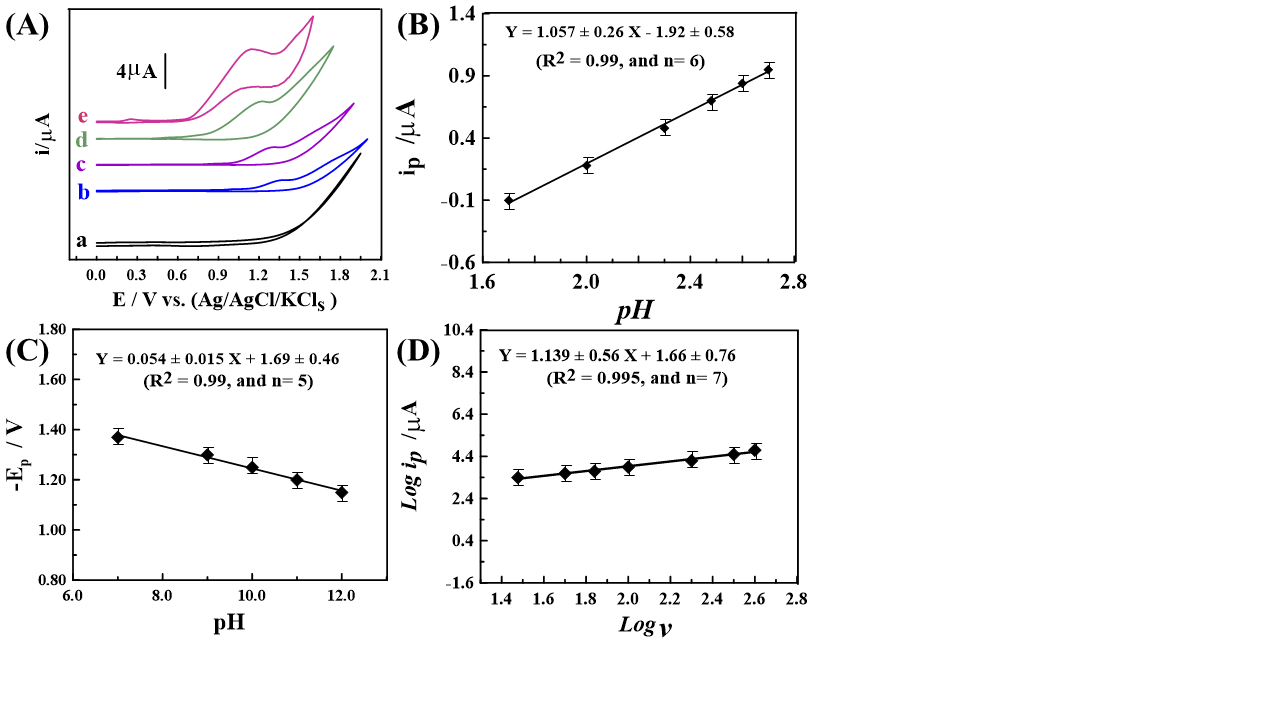
**Fig. S_1_**: The pH_ZPC_ of Co-OF/MMT.

**Fig. S_2_: (A)** CV voltammograms, **(B)** *i*_p_ vs. *pH* plot, **(C)** *E*_p_ vs. *pH* plot, and **(D)** Log i_p_ vs. Log *v* of 1.0 µM CMPA using the 1.0% [Co-OF/MMT] CP sensor at ***v*** = 100 mV·s^−1^.

**
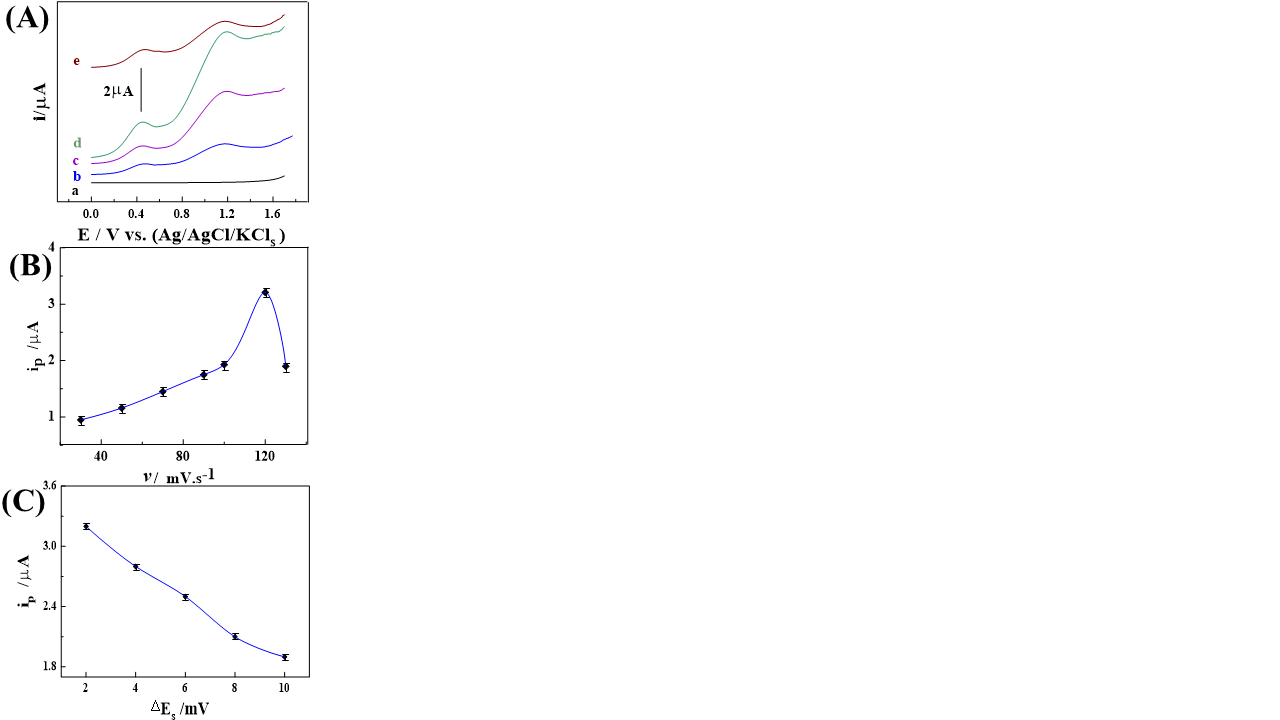
**

**Fig. S_3_:** **(A)** Effect of different PBS buffer with pH of (a) 6, (b) 8, (c) 10, (d) 11, and (e) 12 pH values at 1.0% [Co-OF/MMT] CP sensor (*t_acc_* = 50 s, *E_acc_*= 0.0 V, *v* = 100 mV. S^-1^, and *ΔE_s_* = 2.0 mV). Effect of changing of **(B)** scan rate (*v*), and **(C)** scan increment (*E_s_*) upon the surface of 1.0% [Co-OF/MMT] CP sensor at *E_acc_* = 0.0 V for 50 s.

**Fig. S_4_:** LS-AdASV peaks for (a) 0.3 nM of CMPA (V_I_), (b) 0.3 nM of CMPA spiked with 30.0 nM (~100-fold) of (Mix_1_: Mg^+2^, Zn^+2^, Co^+2^, K^+^, Ca^+2^, Na^+^, and Fe^+2^), and Mix_2_: SO_2_^-2^, PO_4_^-3^, CO_3_^-2^, and Cl^-^), and (c) 0.3 nM of CMPA spiked with 30.0 nM (~100-fold) of GLYP and CPYP on the 1.0% [Co-OF/MMT] CP sensor.
